# Draft Genome Sequence of a *Vibrio harveyi* Strain Associated with Vibriosis in Pacific White Shrimp (*Litopenaeus vannamei*)

**DOI:** 10.1128/genomeA.01662-16

**Published:** 2017-02-16

**Authors:** Emille Moreno, Marci Parks, Lee J. Pinnell, James J. Tallman, Jeffrey W. Turner

**Affiliations:** Department of Life Sciences, Texas A&M University at Corpus Christi, Corpus Christi, Texas, USA

## Abstract

*Vibrio harveyi* is a Gram-negative bacterium associated with vibriosis in penaeid shrimp. Here, we report the draft genome sequence of a *V. harveyi* strain isolated from Pacific white shrimp (*Litopenaeus vannamei*) during a vibriosis outbreak. The availability of this genome will aid future studies of vibriosis in shrimp aquaculture.

## GENOME ANNOUNCEMENT

*Vibrio harveyi* is a Gram-negative marine bacterium and a significant pathogen of marine vertebrates and invertebrates. In the past three decades, coinciding with the rapid growth of shrimp aquaculture, *V. harveyi* has emerged as a significant cause of disease in penaeid shrimp (1). The bacterium is commonly associated with luminous vibriosis, affecting both larvae and adults, and outbreaks have been responsible for mass shrimp mortality, particularly in South America and Asia (2).

We isolated *V. harveyi* strain Hep-2a-10 from Pacific white shrimp (*Litopenaeus vannamei*) during a disease outbreak of unknown etiology. The shrimp were cultured in a zero-exchange, biofloc-dominated, recirculating raceway (40 m^3^) at the Texas AgriLife Research Mariculture Laboratory (Flour Bluff, Corpus Christi, TX, USA). The use of such systems is regarded as more sustainable than traditional pond aquaculture, as they limit the discharge of nutrient-rich effluents (3); however, *Vibrio* outbreaks remain a limiting factor (4).

Samples of hepatopancreas, taken from moribund shrimp, were homogenized, serial-diluted, plated on CHROMagar Vibrio (CHROMagar, Paris, France), and subcultured on thiosulfate-citrate-bile salts-sucrose agar (Oxoid, Hampshire, England). The isolated strain was grown overnight in tryptic soy broth (Becton, Dickinson, Heidelberg, Germany) at 30°C with shaking (100 rpm), and genomic DNA was isolated using a ChargeSwitch gDNA kit per the manufacturer’s instructions (Life Technologies, Inc., Carlsbad, CA, USA). The DNA was quantified using a BioSpectrometer D30 (Eppendorf, Hamberg, Germany) and stored at −20°C. The draft genome of Hep-2a-10 was sequenced, assembled, and annotated as described previously (5). The draft genome was comprised of 67 contigs, with a 44.8% G+C content, and measured 5,917,227 bp. The isolate was confirmed as *V. harveyi* based on 98.62% average nucleotide identity (ANI) with the closed reference genome *V. harveyi* ATCC 43516, as determined by JSpecies (6). Preliminary analysis, conducted using the SEED Viewer (http://theSEED.org), revealed that Hep-2a-10 carries a large repertoire of genes associated with antibiotic resistance, including fluoroquinolone (*n* = 4 genes), tetracycline (*n* = 2 genes), and multidrug (*n* = 28 genes) resistance. Additionally, the genome harbors multiple bacteriocins and genes associated with bile hydrolysis. A more detailed analysis of Hep-2a-10, in comparison with additional outbreak strains, will be the focus of a future publication.

### Accession number(s).

This whole-genome shotgun project has been deposited at DDBJ/ENA/GenBank under the accession number MBTP00000000.

## References

[1] Soto-Rodriguez SA, Gomez-Gil B, Lozano R, del Rio-Rodríguez R, Diéguez AL, Romalde JL 2012 Virulence of *Vibrio harveyi* responsible for the “Bright-red” syndrome in the Pacific white shrimp *Litopenaeus* *vannamei*. J Invertebr Pathol 109:307–317. doi:10.1016/j.jip.2012.01.006. 22306693

[2] Austin B, Zhang XH 2006 *Vibrio harveyi*: a significant pathogen of marine vertebrates and invertebrates. Lett Appl Microbiol 43:119–124. doi:10.1111/j.1472-765X.2006.01989.x. 16869892

[3] Krummenauer D, Samocha T, Poersch L, Lara G, Wasielesky W Jr. 2014 The reuse of water on the culture of Pacific white shrimp, *Litopenaeus* *vannamei*, in BFT System. J World Aquacult Soc 45:3–14. doi:10.1111/jwas.12093.

[4] Prangnell DI, Castro LF, Ali AS, Browdy CL, Zimba PV, Laramore SE, Samocha TM 2016 Some limiting factors in Superintensive production of juvenile pacific white shrimp, *Litopenaeus* *vannamei*, in No-water-exchange, Biofloc-dominated systems. J World Aquacult Soc 47:396–413. doi:10.1111/jwas.12275.

[5] Collin B, Pinnell LJ, Tallman JJ, Turner JW 2016 Draft genome sequences of one marine and one clinical vibrio parahaemolyticus strain, both isolated in Sweden. Genome Announc 4(5):e01196-16. doi:10.1128/genomeA.01196-16. 27789643 PMC5084867

[6] Richter M, Rosselló-Móra R 2009 Shifting the genomic gold standard for the prokaryotic species definition. Proc Natl Acad Sci U S A 106:19126–19131. doi:10.1073/pnas.0906412106. 19855009 PMC2776425

